# Identification of AnnexinA1 as an Endogenous Regulator of RhoA, and Its Role in the Pathophysiology and Experimental Therapy of Type-2 Diabetes

**DOI:** 10.3389/fimmu.2019.00571

**Published:** 2019-03-27

**Authors:** Gareth S. D. Purvis, Massimo Collino, Rodrigo A. Loiola, Andrea Baragetti, Fausto Chiazza, Martina Brovelli, Madeeha H. Sheikh, Debora Collotta, Alessia Cento, Raffaella Mastrocola, Manuela Aragno, Juan C. Cutrin, Chris Reutelingsperger, Liliana Grigore, Alberico L. Catapano, Magdi M. Yaqoob, Giuseppe Danilo Norata, Egle Solito, Christoph Thiemermann

**Affiliations:** ^1^Department of Translational Medicine and Therapeutics, Bart's and The London School of Medicine and Dentistry, The William Harvey Research Institute, Queen Mary University of London, London, United Kingdom; ^2^Department of Drug Science and Technology, University of Turin, Turin, Italy; ^3^Department of Pharmacological and Biomolecular Sciences, Università Degli Studi di Milano, Milan, Italy; ^4^Centro SISA per lo studio del'Aterosclerosi, Bassini Hospital, Lombardy, Italy; ^5^Department of Clinical and Biological Sciences, University of Turin, Turin, Italy; ^6^Department of Molecular Biotechnology and Sciences for the Health, University of Turin, Turin, Italy; ^7^Department of Biochemistry, Cardiovascular Research Institute, Maastricht University, Maastricht, Netherlands; ^8^IRCCS Multimedica, Lombardy, Italy; ^9^Dipartimento di Medicina Molecolare e Biotecnologie Mediche, Università Degli Studi di Napoli “Federico II”, Naples, Italy

**Keywords:** type-2 diabetes, metabolism, Annexin A1, nephropathy, hepatosteatosis, Rho A

## Abstract

Annexin A1 (ANXA1) is an endogenously produced anti-inflammatory protein, which plays an important role in the pathophysiology of diseases associated with chronic inflammation. We demonstrate that patients with type-2 diabetes have increased plasma levels of ANXA1 when compared to normoglycemic subjects. Plasma ANXA1 positively correlated with fatty liver index and elevated plasma cholesterol in patients with type-2 diabetes, suggesting a link between aberrant lipid handling, and ANXA1. Using a murine model of high fat diet (HFD)-induced insulin resistance, we then investigated (a) the role of endogenous ANXA1 in the pathophysiology of HFD-induced insulin resistance using ANXA1^−/−^ mice, and (b) the potential use of hrANXA1 as a new therapeutic approach for experimental diabetes and its microvascular complications. We demonstrate that: (1) ANXA1^−/−^ mice fed a HFD have a more severe diabetic phenotype (e.g., more severe dyslipidemia, insulin resistance, hepatosteatosis, and proteinuria) compared to WT mice fed a HFD; (2) treatment of WT-mice fed a HFD with hrANXA1 attenuated the development of insulin resistance, hepatosteatosis and proteinuria. We demonstrate here for the first time that ANXA1^−/−^ mice have constitutively activated RhoA. Interestingly, diabetic mice, which have reduced tissue expression of ANXA1, also have activated RhoA. Treatment of HFD-mice with hrANXA1 restored tissue levels of ANXA1 and inhibited RhoA activity, which, in turn, resulted in restoration of the activities of Akt, GSK-3β and endothelial nitric oxide synthase (eNOS) secondary to re-sensitization of IRS-1 signaling. We further demonstrate in human hepatocytes that ANXA1 protects against excessive mitochondrial proton leak by activating FPR2 under hyperglycaemic conditions. In summary, our data suggest that (a) ANXA1 is a key regulator of RhoA activity, which restores IRS-1 signal transduction and (b) recombinant human ANXA1 may represent a novel candidate for the treatment of T2D and/or its complications.

## Introduction

Obesity and metabolic syndrome are of global health concern and are an independent risk factor of diseases characterized by systemic inflammation such as type-2 diabetes mellitus (T2D), non-alcoholic fatty liver disease (NAFLD/NASH) (1), chronic kidney disease (CKD) (2), and cardiovascular disease (3). Even with aggressive strategies to modulate both plasma lipid profiles and blood glucose levels, microvascular complications develop over time in patients with T2D (4). Indeed, ~30% of patients with T2D develop diabetic nephropathy, which is the leading cause of end-stage renal disease (ESRD). Peripheral insulin resistance drives a vicious pathophysiological cycle leading to T2D, ectopic adiposity and chronic inflammation, coupled with a down-regulation of pro-survival/anti-inflammatory pathways. Identifying endogenous molecules that are both anti-inflammatory and tissue protective agents will lead to the discovery of novel drug target for the treatment of diabetes.

Annexin A1 (ANXA1) is an endogenous anti-inflammatory protein, principally known as a regulator of peripheral leukocyte migration and a promoter of macrophage phagocytosis of apoptotic neutrophils (5). ANXA1 is expressed by endothelial cells, tubular epithelial cells, adipocytes, and low levels of ANXA1 can be detected in the circulation under physiological conditions. We recently demonstrated that the plasma levels of ANXA1 are elevated in patients with type-1 diabetes (6) and multiple sclerosis (7). Cristante et al. (7) demonstrated a mechanistic link between RhoA and ANXA1, whereby ANXA1 interacts with RhoA to alter actin-polymerization and maintain blood brain barrier integrity. RhoA has far reaching implications in many diseases including type-2 diabetes, its activity is upregulated by oxidative stress and hyperglycemia. Many strategies, which inhibit the Rac/RhoA pathway, also reduce microvascular complications in diabetes via a reduction in inflammation and fibrosis (8, 9).

In the present study, we use a translational approach to gain a better insight into the role of ANXA1 in (a) patients with T2D and (b) a murine model of high fat diet (HFD) induced insulin resistance. Moreover, we investigated the effect of human recombinant ANXA1 (hrANXA1) as a potential treatment for experimental T2D and its microvascular complications.

## Methods

### Human Studies and Subjects and Ethic Statement

Human volunteers and patients were recruited within the general population enrolled in the PLIC study (Progressione delle Lesioni Intimali Carotidee) at the Center for the Study of Atherosclerosis, SISA Bassini Hospital Cinisello B. Italy (Ethical approval SEFAP/Pr0003F University of Milan 06/2/2001) (10). Patients with T2D were identified from their clinical history, outpatient's registries and/oral hospital archives, following international guidelines (11). Subjects included in the study were defined as “normoglycemic” if the following criteria were met: No self-reported T2D, patients are not being treated with glucose lowering drugs and fasting glucose levels are below 110 mg/dL (at least 3 previous controls). All patients and healthy volunteers gave written informed consent in adherence to the Declaration of Helsinki. Clinical data was collected, and biochemical analyses were performed as previously described (12). Urinary albumin levels were determined by immunoturbidimetry on fresh samples of the morning. Fatty Liver Index was calculated from Body Mass Index (BMI), waist circumference, gamma-glutamyl-transpeptidase (GGT) and triglyceride levels in fasting condition as previously described (13).

Healthy subjects” from the PLIC cohort were only included if they had no evidence of hepatic steatosis (ultrasound determined, not shown), renal damage or type-2 diabetes (T2D). Clinical information [clinical and pharmacological history, BMI, and waist-hip ratio (waist)] was collected during the outpatient activity, which was part of the study design of PLIC. Biochemical, lipid profile (LDL-C “LDL cholesterol levels”), liver enzymes (including gamma-glutamyl-transferase), C-reactive protein (CRP) and glucose level were determined as previously described (12). Briefly, blood samples were drawn after overnight fasting (10 h at least) from antecubital vein and collected in EDTA tubes (BD Vacuette®). Blood samples were then centrifuged at 3,000 rpm for 12 min in order to separate plasma for glucose quantification. Determination was performed by enzymatic method (hexokinase reaction) through automatic sample analyzer (RX Daytona, Randox Laboratories Ltd®, Crumlin, UK).

Fatty liver Index (FLI) was determined as previously described, according to the following formula: FLI = (e^0.953^*loge(*triglycerides*) +0.139^*^BMI + 0.718^*^loge(*GGT*)+0.053^*^waistcircumference−15.745)/(1+*e*^0.953*^loge(*triglycerides*)+0.139^*^BMI+0.718*loge(*GGT*)+0.053^*^waistcircumference−15.745)^*^ 100.

Chronic kidney disease (CKD) and its stages (CKD3, CKD4, and CKD5) were determined following determination of glomerular filtration rate (GFR), according to validated international criteria. When compared to healthy controls, CKD patients had a GFR of <60 ml/min/1.73 m^2^ associated with albuminuria (urinary albumin over 30 mg/g of total urinary proteins).

### Use of Experimental Animals-Ethics Statement

The experimental protocols used in this study have been approved by the Animal Welfare Ethics Review Board (AWERB) of Queen Mary University of London and the University of Turin, the study was performed under license issued by the home office (Procedure Project License; PPL: 70/8052) and committees (DGSAF 0021573-P-12/11/2013 and DGSAF). Animal care was in accordance with the Home Office guidance on Operation of Animals (Scientific Procedures Act 1986) published by Her Majesty's Stationery Office and the Guide for the Care and Use of Laboratory Animals of the National Research Council and are in keeping with the European Directive (2010/63/EU) as well as the Guide for the Care and Use of Laboratory Animals.

### Animals and Experimental Procedures

This study was carried out on 10 weeks old ANXA1^−/−^ mice on a C57BL/6 background (14) and wild-type (WT) C57BL/6 mice, housed in the same unit under conventional housing conditions at 25 ± 2°C. WT and ANXA1^−/−^ mice were randomly assigned either normal diet (chow) or high fat, high sugar diet (HFD) (D12331diet, Research Diet Inc., USA). All mice had access to food and water *ad libitum*. After 4 weeks of dietary manipulation, mice were randomly assigned to a treatment group receiving either with hrANXA1 (40 μg/kg, i.p.) or vehicle (Hepes 50 mM, NaCl 140 mM i.p.) 5 days per week for 6 weeks. Mice were harvested the morning after receiving the last dose of hrANXA1 (day 5 of week 6 of treatment). hrANXA1 was produced and purified as previously published, ~0.5% of injected dose per gram (ID/g) ANXA1 remained in the circulation 24 h post injection as previously reported (15).

### ELISA for ANXA1

A homemade sandwich ELISA was used to measure plasma ANXA1 (16). Briefly, ELISA-treated plates (Nunc MaxiSorp, ThermoScientific, UK) were incubated overnight with capture antibody 20 μg/ml (mouse monoclonal antibody, generated in house) in bicarbonate buffer (25 mM NaHCO_3_, 25 mM Na2CO_3_, pH 9.6). The plate was then washed 3 times with bicarbonate buffer and blocked in blocking buffer (0.1% BSA, PBS) for 1 h at 37°C. Then 100 μl of sample and standard in assay diluent (Tween-20 0.05% (v/v), PBS) were loaded and incubated for 1 h at 37°C, then washed 5 times with wash buffer (0.9% (w/v) NaCl, 0.05% (v/v) Tween-20, dH_2_O). Following this wells were incubated with 1 μg/ml of detecting antibody (rabbit polyclonal anti-ANXA1; Invitrogen, UK) for 1 h at 37°C. After 5 washes, immuno-complexes were detected by adding the goat-anti-rabbit IgG with conjugated alkaline phosphatase for 30 min. After 5 washes, the substrate, p-nitrophenyl phosphate (Sigma Aldrich, UK) was added and left for 30 min for full development of color. The plate was then read absorbance at 405 nm and corrected at 540 nm as a reference wavelength, using a spectrofluorometer (Tecan Infinite M200 Pro, Tecan, Austria).

### Oral Glucose Tolerance test (OGTT)

Mice were fasted for 6 h prior to testing, then given an oral bolus of glucose (2 g/kg in H_2_O p.o.). Blood glucose was measured from the tail vein at time 0 and then at 15 min intervals for 120 min using glucometer (Accu-Chek Compact System, Roche Diagnostics); basal non-fasted blood glucose.

### Blood and Biochemical Analysis

Serum triglyceride and total cholesterol were measured by standard enzymatic assay using reagent kits (Hospitex Diagnostics, Italy). Liver triglycerides were measured via a colorimetric assay (Abnova Corporation, Germany). ALT and urine and serum creatinine were measured by a commercial veterinary testing laboratory (IDEXX, Wetherby, UK); serum insulin and urine albumin were measured using commercially available ELISA kits (Abcam, Cambridge, UK, and Bethyl Laboratories, Montgomery, TX, USA).

### Histological Analysis

#### Oil Red-O

Frozen liver samples were embedded in OCT, and cut in 10 μm sections. Section were brought to room temperature, fixed with 10% buffer formalin for 5 min, washed with 60% isopropanol, then saturated with Oil Red O (1% w/v, 60% isopropanol) for 15 min, washed in 60% isopropanol and rinsed in distilled water. The sections were then mounted in aqueous mounting medium with coverslips. Images were acquired using a NanoZoomer Digital Pathology Scanner (Hamamatsu Photonics K.K., Japan) and analyzed using the NDP Viewer software. Additionally 10 randomly selected fields of view from each liver section were used to assess lipid accumulation.

#### Periodic Acid Schiff's

Kidney samples were obtained at the end of the experiment and fixed in 10% neutral-buffered formalin for 48 h and histology staining was performed. Briefly, kidney tissue was embedding in paraffin and processed to obtain 4 μm sections. After deparaffinization and sections were rehydrated through graded alcohol to distilled water. The sections were then incubated with saturated in Periodic Acid Schiffs (Sigma, UK) solution for 30 min and washed in distilled water. Then sections were then dehydrated through graded alcohols and cleared before mounting with coverslips. Images were acquired using a NanoZoomer Digital Pathology Scanner (Hamamatsu Photonics K.K., Japan) and analyzed using the NDP Viewer software. Additionally 10 randomly selected fields of view from each kidney section were used to assess structural alteration of the proximal convoluted tubules and general renal histopathology.

### Western Blot Analysis

Semi-quantitative western blot analyses of phosphorylated and/or total form of IRS-1, Akt, GSK-3β, endothelial nitric oxide synthase (eNOS), ANXA1, RhoA, and MYPT1 were carried out in tissue samples as described before (6). Briefly, liver, skeletal muscle, and kidney samples were homogenized in protein homogenization buffer and centrifuged at 1,300 g for 5 min at 4°C. To obtain the cytosolic protein fraction, supernatants were centrifuged at 16,000 g at 4°C for 40 min. Protein content was determined on cytosolic extracts using bicinchoninic acid (BCA) protein assay (Thermo Fisher Scientific, Rockford, IL). Proteins were separated by 8% sodium dodecyl sulfate polyacrylamide gel electrophoresis (SDS-PAGE) and transferred to a polyvinyldenediflouoride (PVDF) membrane, which were blocked with a solution of 5% dry milk in TBS-Tween for 2 h. Membranes were incubated with a primary antibody (1:1,000 rabbit anti-total IRS-1; 1:1,000 rabbit anti-pSer^307^ IRS-1; 1:1,000 rabbit anti-total Akt; 1:1,000 rabbit anti pSer^473^ Akt; 1:1,000 rabbit anti-total GSK−3β 1:1,000 rabbit anti-pSer^9^GSK−3β; 1:200 rabbit anti-ANXA1; 1:1,000 rabbit anti-total RhoA; 1:1,000 rabbit anti-pSer^188^ RhoA; 1:1,000 rabbit anti-total MYPT1 and 1:1,000 rabbit anti-pSer^853^ MYPT1). Membranes were incubated with a secondary antibody conjugated with horseradish peroxidase (1:2,000) for 30 min at room temperature and developed with ECL detection system. The immunoreactive bands were visualized by autoradiography and the densitometry analysis was performed using Gel Pro Analyser 4.5, 2,000 software (Media Cybernetics, Silver Spring, MD, USA). The membranes were stripped and incubated with alpha tubulin monoclonal antibody (1:5,000) and subsequently with an anti-mouse antibody (1:2,000) to assess gel-loading homogeneity. Densitometry analysis of the related bands is expressed as relative optical density, and normalized using the related WT + vehicle or sham band.

### Oxygen Consumption Rate

HepG2 cells (3 × 10^5^) were seeded in 96-well Seahorse plates in serum free medium containing 5.5 mM glucose, 25 mM glucose, 25 mM glucose + hrANXA1 (20 μg/mL) or 25 mM glucose + hrANXA1 (20 μg/mL) + WRW4 an FPR2 antagonist [0.9 mM-Tocris- inhibits WKYMVm binding to FPR2 (IC_50_ = 0.23 μM)] and incubated for 48 h. to measure OCR, the medium was replaced with XF Mitoassay medium (pH 7.4) and incubated without CO_2_ for 30 min. Then, basal OCR was measured and wells were sequentially injected with: (1) oligomycin (1.0 μM), an ATP synthase blocker; (2) carbonyl cyanide p-[trifluoromethoxy]-phenyl-hydrazone (FCCP) (0.5 μM), a proton ionophore; and (3) a mix of rotenone (0.5 μM) and antimycin A (0.5 μM), inhibitors of electron transport. OCR was measured three times following each injection with an interval of 6 min between each reading. Experiments were performed three times in triplicate and all values of OCR were normalized to protein content of individual wells. Data was gathered on SeaHorse XFe96 Analyzer (Agilent Technologies) and data analyzed using Wave Software (Agilent Technologies).

### Image Stream

HepG2 cells were seeded into 6-well plates at a density of 1 × 10^6^ and then treated with DMEM containing glucose (5.5 or 25 mM). After 48 h, cells were harvested and fixed in paraformaldehyde (2%) for 10 min at room temperature and washed in PBS. Cells were then blocked in blocking solution (PBS + 0.2% BSA) for 30 min at room temperature. To perform the staining of FPR2 on the membrane, cells were then incubated with primary anti-body [rabbit anti-FPR2 (1:50)] (Acris Antibodies) (30 min at room temperature), followed by incubation with secondary anti-body [anti-rabbit AF488 (1:100)] (30 min at room temperature). From intracellular FPR2 and ANXA1 cells were permeabilized with permeabilization solution (PBS + Tween20 0.5% v/v, 10 min at room temperature); cells were then washed and blocked in blocking solution (PBS + 0.2% BSA, 30 min at room temperature). Cells were then incubated with primary anti-body [rabbit anti-FPR2 (1:50)] (Acris Antibodies) and mouse anti-ANXA1 (Invitrogen (1:100) washed and incubated with secondary anti-body (anti-rabbit AF647 (1:100) and anti-mouse AF405 (1:100) (30 min at room temperature), and then washed. Imaging flow cytometry was performed on an ImageStreamx Mark II operated by INSPIRE software (Amnis Corporation). A sample of HEPG2 that were not incubated with antibodies were collected at the same settings, in order to gate different cell populations (negative or positive staining). In each experiment, a template of settings was created and it was applied to all files. A total of 10,000 events were collected for each sample, and data were analyzed using IDEAS Application 6.1 software (Amnis Corporation).

### Human Phospho-Kinase Array

HepG2 cells were incubated in DMEM containing 5.5, 25, and 25 mM glucose + hrANXA1 (20 μg/mL) or 25 mM glucose + hrANXA1 (20 μg/mL)+ WRW4 (0.9 mM) and incubated for 48 h. Cells were washed in PBS containing protease inhibitors and cell lysates extracted as per manufactures instructions. Protein concentration was quantified and 600 μg of protein was used per membrane of a Proteome Profiler, Human Phospho-kinase Array (R&D Systems). After visualization of the spots, the signals were measured using Image Studio LICOR, normalization was carried as per manufacturers instruction and changes plotted in a heat map expressed as fold change to 5.5 mM glucose for each individual phosphorylation site using PRISM.

### Statistical Analysis

**Figure 1:**
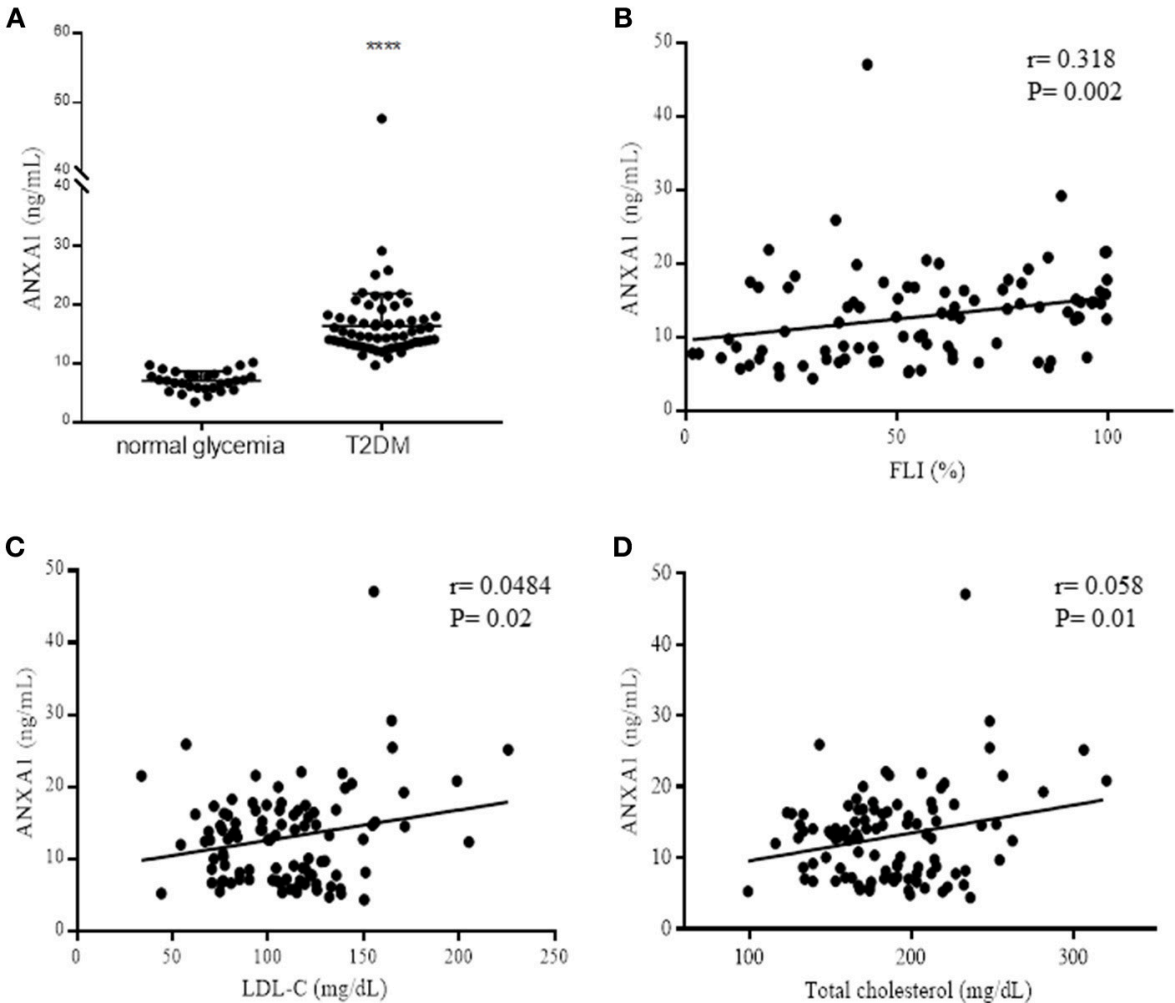
Assessment of ANXA1 levels in patients with type-2 diabetes. **(A)** Plasma ANXA1 levels measured by ELISA in age and sex match normoglycemic (*n* = 30) and patients with type-2 diabetes (*n* = 65). **(B)** Correlation in diabetic patients of plasma ANXA1 levels and: Fatty Liver Index **(B)**, LDL-C **(C)**, and total cholesterol **(D)**. Data is expressed as mean± SEM., ^****^p < 0.0001. **(B–D)** 95% confidence intervals are displayed of the microvascular and significance estimated using Fishers *F*-test *p* < 0.05 was deemed significant.

**Figure 2:**
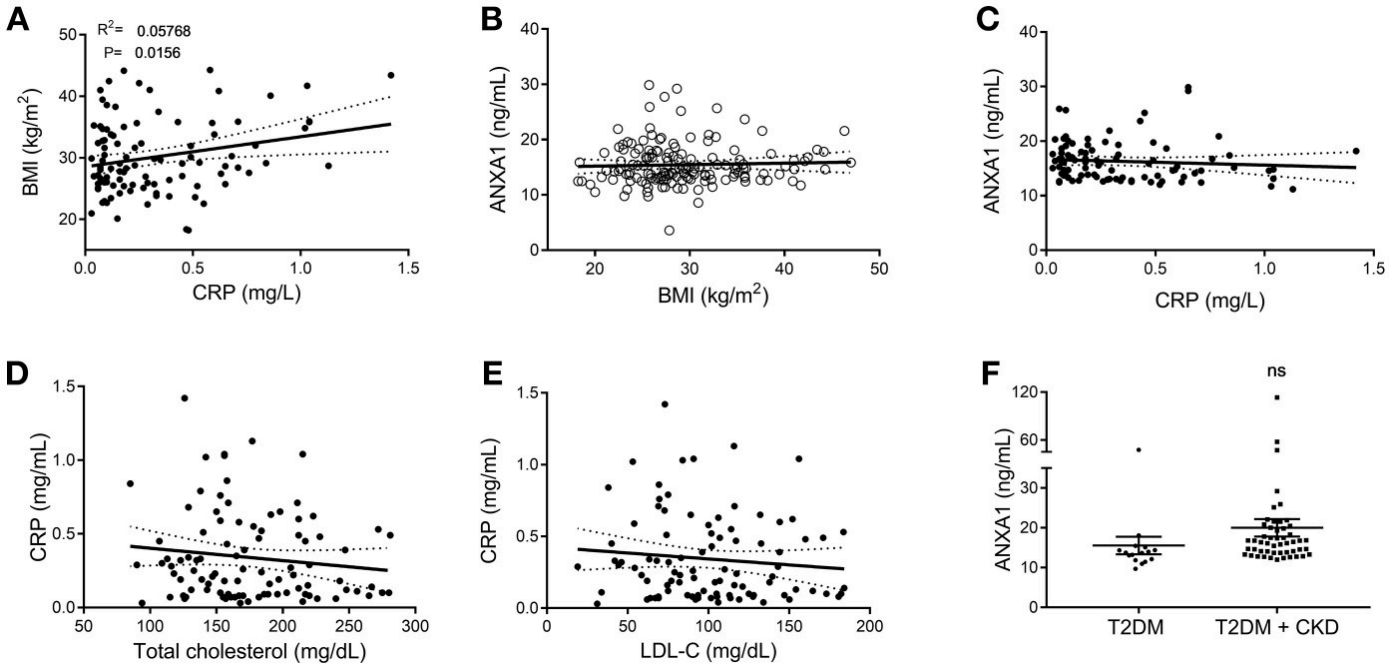
Correlation data of clinical markers in patients with type-2 diabetes and ANXA1. **(A)** Correlation of plasma CRP with BMI in diabetic patients. **(B)** Correlation of plasma ANXA1 levels with BMI in diabetic patients. **(C)** Correlation of plasma ANXA1 levels with CRP in diabetic patients. **(D)** Correlation of CRP with cholesterol in diabetic patients. **(E)** Correlation of CRP with LDL in diabetic patients. **(F)** Plasma ANXA1 levels in patients with diabetes ± CKD. **(A–E)** display 95% confidence intervals are displayed of the linear regression and significance estimated using Fishers *F*-test *p* < 0.05 was deemed significant.

**Figure 3:**
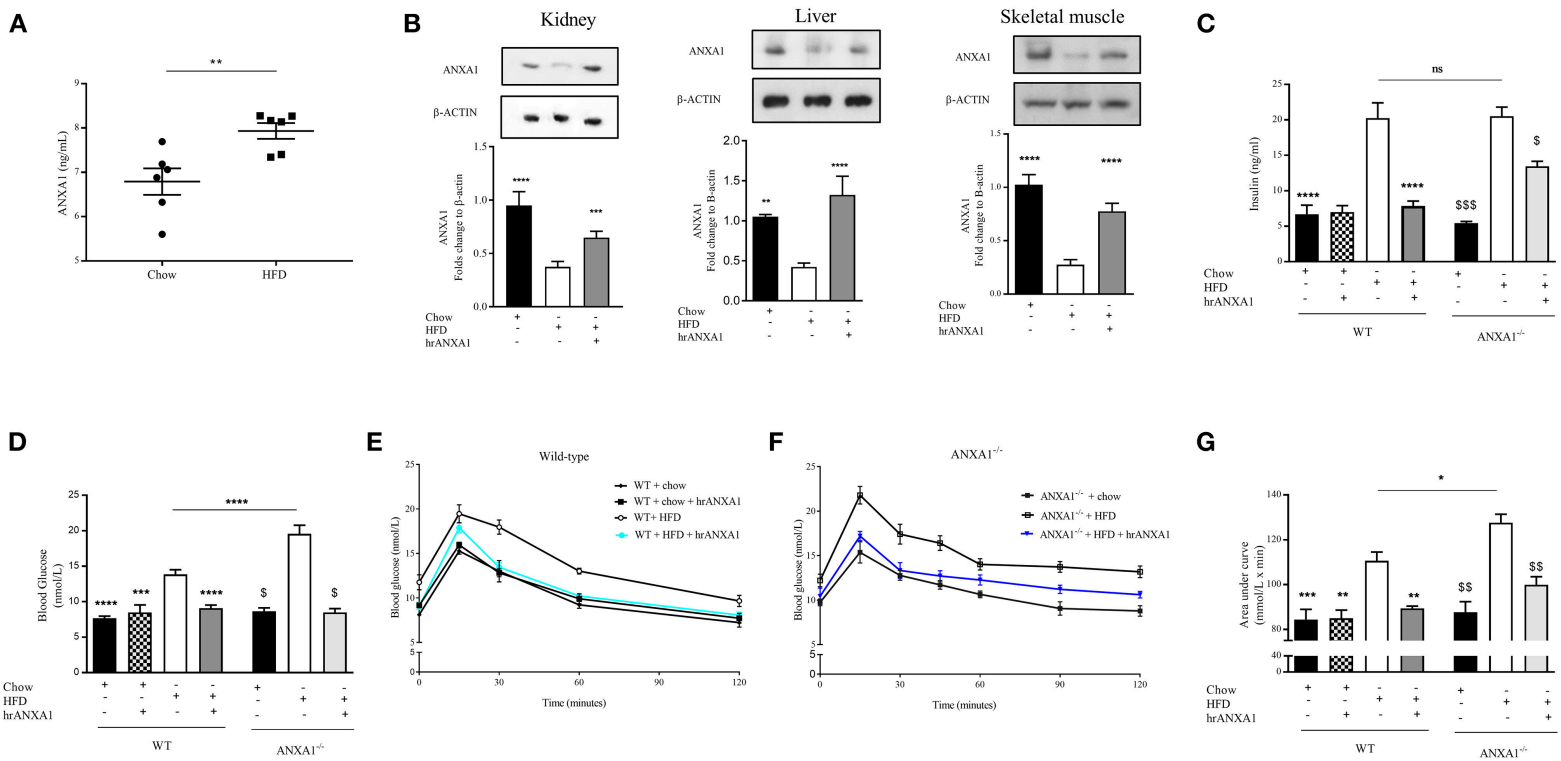
ANXA1 attenuates the development of obesity and insulin resistance in HFD fed mice. C57BL/6 or ANXA1^−/−^ mice, fed a standard diet (chow) or a high-fat diet (HFD) for 10 weeks, were treated with vehicle or human recombinant (hr) ANXA1 (40 μg/kg, i.p.) five times per week between weeks 5 and 10. **(A)** ELISA for ANXA1 levels were measured in serum isolated from whole blood at harvest, (*n* = 6) **(B)** Western blot analysis of kidney, liver, and skeletal muscle show a depletion in ANXA1 which was restored by hrANXA1 treatment (*n* = 6/group). **(C)** Serum insulin levels were measured in plasma isolated from whole blood at harvest (*n* = 6–10/group). **(D)** Basal non-fasted blood glucose was measured at week 10 1 h prior to harvest via the tail vain (*n* = 6–10/group). **(E)** Oral glucose tolerance was assessed over 120 min 1 week prior to harvest in WT mice. **(F)** Oral glucose tolerance was assessed over 120 min 1 week prior to harvest in ANXA1^−/−^ mice. **(G)** The area under curve (AUC) of OGTT was calculated for respective groups and used for statistical analysis. Data analyses by a one-way ANOVA followed by a Bonferroni *post-hoc* test and the mean is expressed mean ± SEM., ^*^p < 0.05, ^**^p < 0.01, ^***^p < 0.001, ^****^p < 0.0001; ^$^*p* < 0.05 vs. WT + HFD; and ^*$$*^*p* < 0.01, ^*$$$*^*p* < 0.001 vs. ANXA1^−/−^ + HFD.

**Figure 4:**
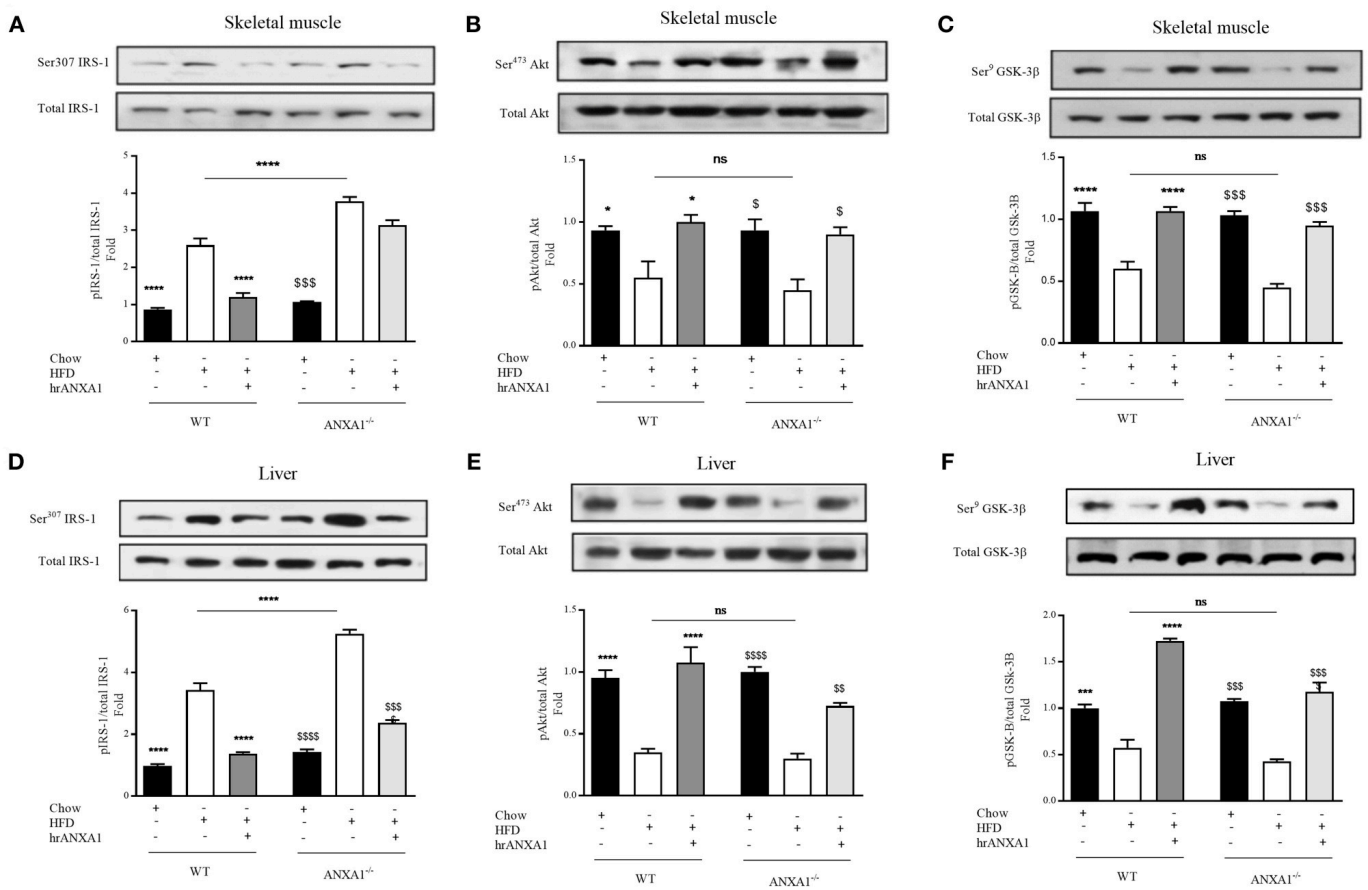
ANXA1 attenuates HFD induced development of peripheral insulin resistance. C57BL/6 or ANXA1^−/−^ mice, fed a standard diet (chow), or a high-fat diet (HFD) for 10 weeks, were treated with vehicle or human recombinant (hr) ANXA1 (40 μg/kg, i.p.) five times per week between weeks 5 and 10. Western blot analysis for Phosphorylation of Ser^307^ on IRS-1 in Skeletal muscle **(A)** or liver **(D)** and normalized to total IRS-1; for phosphorylation of Ser^473^ on Akt in the skeletal muscle **(B)** and liver **(E)** and normalized to total Akt; for phosphorylation of Ser^9^ on GSK-3β in the skeletal muscle **(C)** and in the liver **(F)** and normalized to total GSK-3β. Data were analyzed by a one-way ANOVA followed by a Bonferroni *post-hoc* test and the mean is expressed mean ± SEM., *p < 0.05, ^***^p < 0.001, ^****^p < 0.0001 vs. WT + HFD; ^$^*p* < 0.05, ^*$$*^*p* < 0.01, ^$$$^*p* < 0.001, ^$$$$^*p* < 0.0001 vs. ANXA1^−/−^ +HFD.

**Figure 5:**
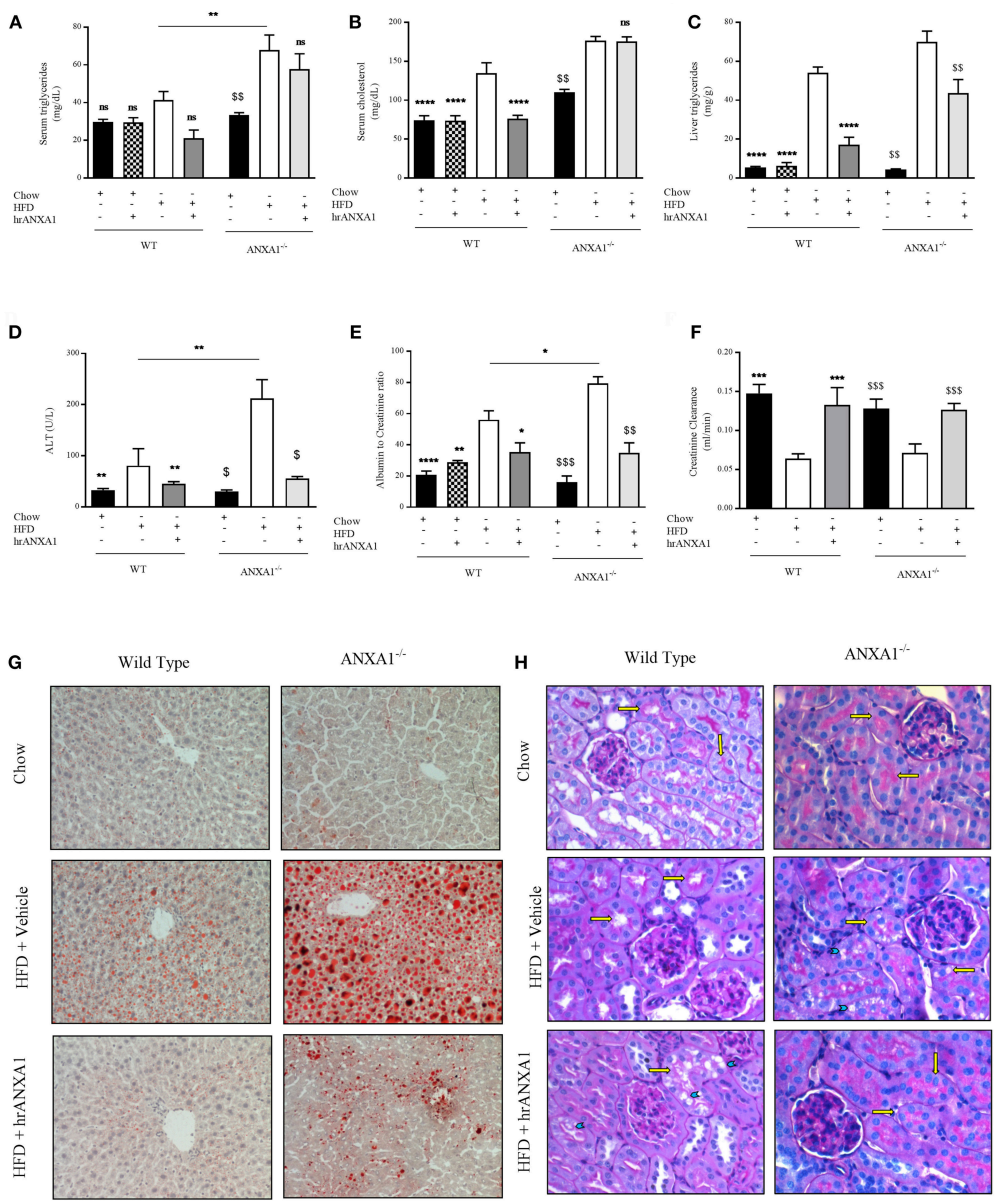
ANXA1 attenuates induced lipid accumulation, hepatic injury, and renal dysfunction. C57BL/6 or ANXA1^−/−^ mice, fed a standard diet (chow) or a HFD for 10 weeks, were treated with vehicle or human recombinant (hr) ANXA1 (40 μg/kg, i.p.) five times per week between weeks 5 and 10. Measure of **(A)** Serum triglyceride, **(B)** Serum cholesterol, **(C)** Liver triglyceride, **(D)** Serum aminotransferase (ALT), *n* = 6–8/group. **(E)** 18 h urine samples was collected and renal dysfunction was measured by albumin to creatinine ratio (ACR), *n* = 6–8 per group. **(F)** Creatinine clearance was measured from urinary and serum creatinine, *n* = 6–8 per group. **(G)**: representative images of hepatic lipid deposition assessed by Oil Red-O staining. Panel **(H)**: representative images of histological changes in kidney structure assessed by periodic acid-Schiff staining, yellow arrows indicating brush borders of proximal tubules. Data were analyzed by a one-way ANOVA followed by a Bonferroni *post-hoc* test and the mean is expressed mean ± SEM., ^*^p < 0.05, ^**^p < 0.01, ^***^p < 0.001, ^****^p < 0.0001 vs. WT + HFD. ^$^*p* < 0.05, ^$$^*p* < 0.01, ^$$$^*p* < 0.001 vs. ANXA1^−/−^ + HFD.

**Figure 6:**
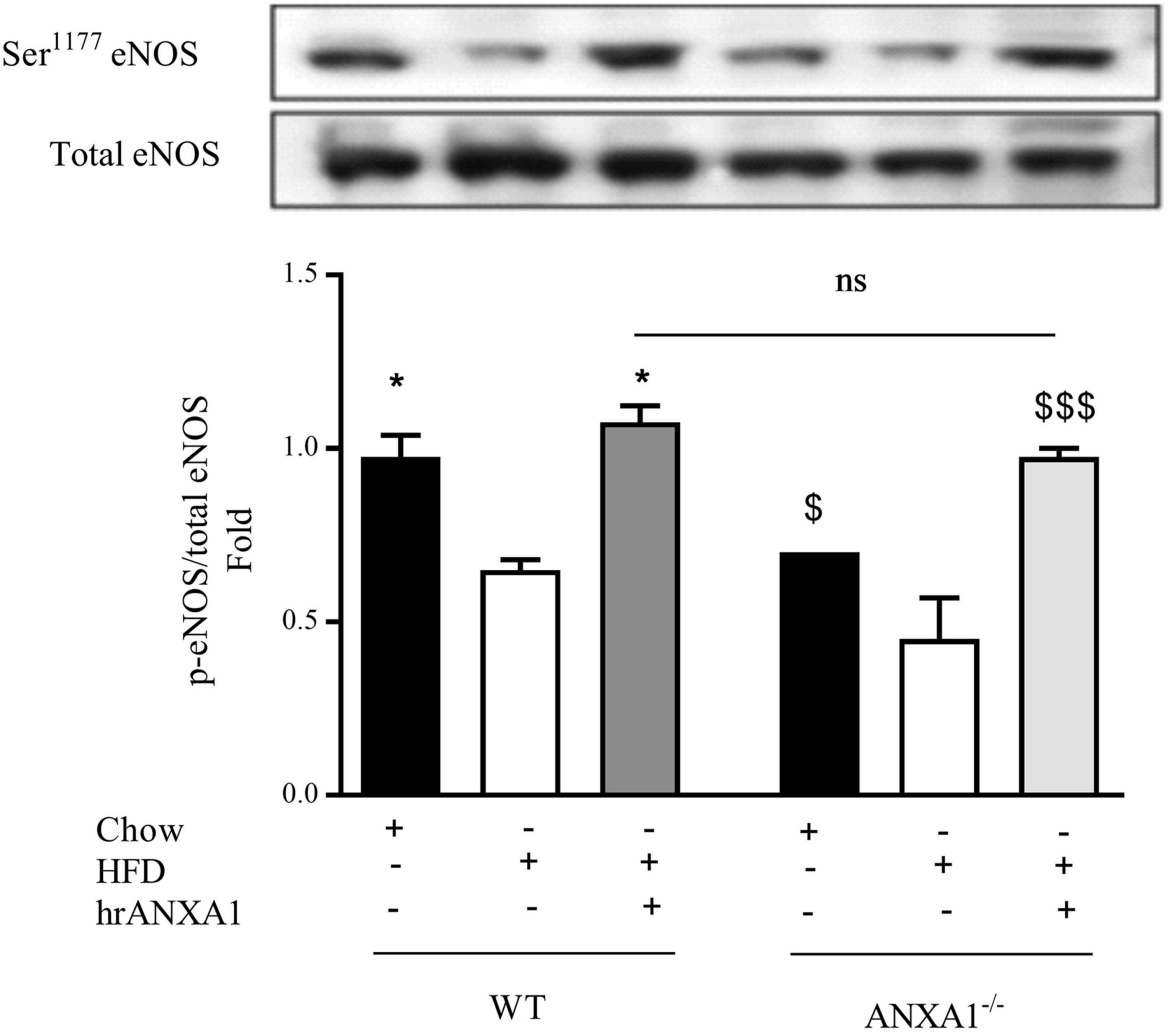
ANXA1 attenuates HFD induction of eNOS in the diabetic kidney C57BL/6 or ANXA1^−/−^ mice, fed a standard diet (chow), or a high-fat high-sugar diet (HFD) for 10 weeks, were treated with vehicle or hrANXA1 (40 μg/kg, i.p.) five times per weeks between weeks 5 and 10. At harvest kidney, liver and skeletal muscle was harvested. Phosphorylation of eNOS was determined by western blot; a representative blot is shown and densitometry quantification of *n* = 3 independent experiments. ^*^p < 0.05 and ^*$*^*p* < 0.05, ^*$$$*^*p* < 0.001.

**Figure 7:**
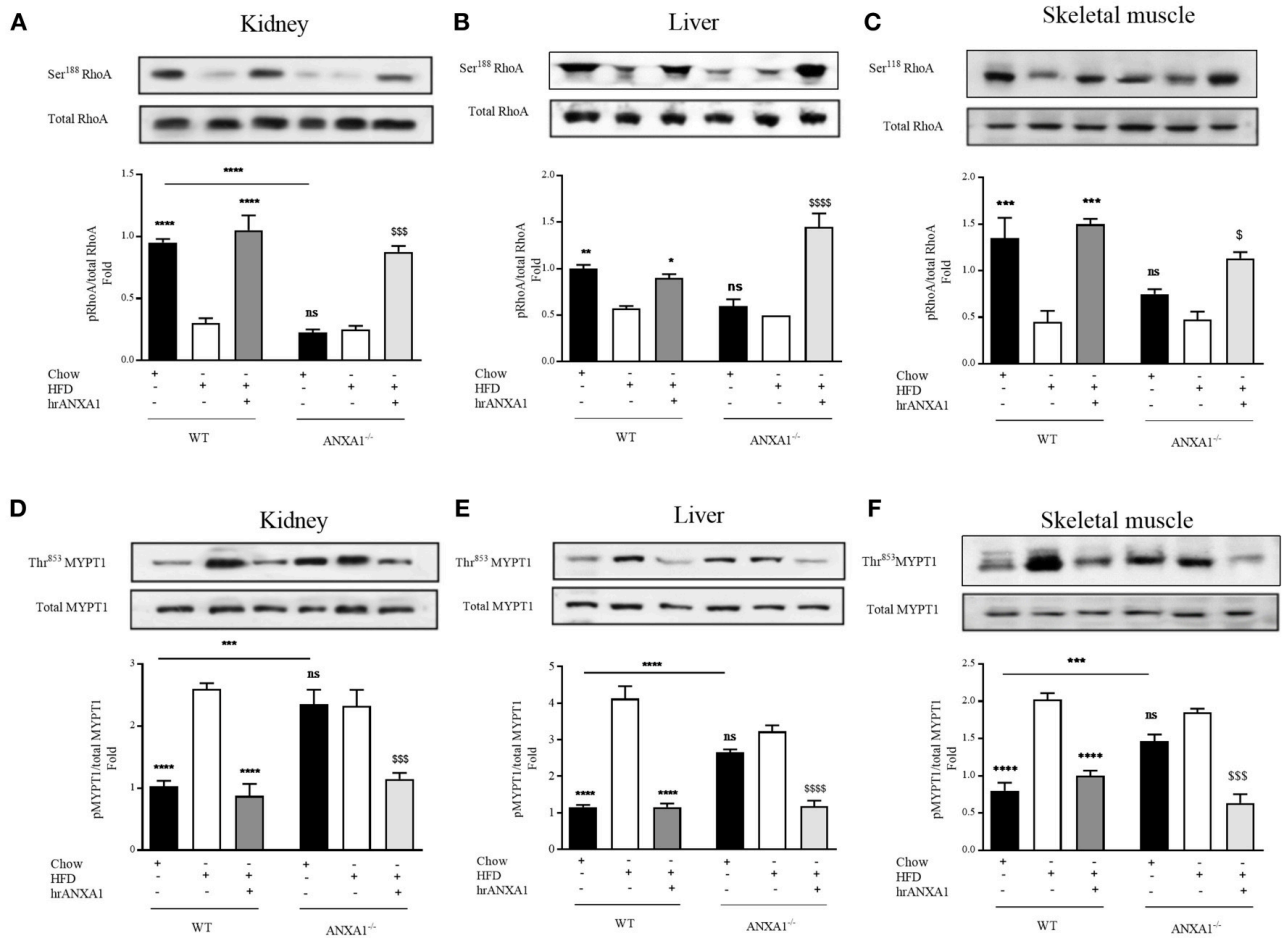
ANXA1 attenuates RhoA induction in mice fed a HFD. C57BL/6 or ANXA1^−/−^ mice, fed a standard diet (chow) or a high-fat high-sugar diet (HFD) for 10 weeks, were treated with vehicle or hrANXA1 (40 μg/kg, i.p.) five times per weeks between weeks 5 and 10. At harvest kidney, liver and skeletal muscle was collected. **(A–C)** Phosphorylation of Ser^188^ on RhoA was normalized to total RhoA in kidney **(A)**, liver **(B)**, and skeletal muscle **(C)**. **(D–F)** Phosphorylation of Thr^853^ on MYPT1 was normalized to total MYPT1 in kidney **(D)**, liver **(E)**, and skeletal muscle **(F)**. A representative blot is shown for each protein and densitometry quantification of *n* = 3 experiments represented in the histograms. Data were analyzed by a one-way ANOVA followed by a Bonferroni *post-hoc* test and the mean is expressed mean ± SEM., ^*^p < 0.05, ^**^p < 0.01, ^***^p < 0.001, ^****^p < 0.0001 vs. WT + HFD; ^$^*p* < 0.05, ^$$$^*p* < 0.001, ^$$$$^*p* < 0.0001 vs. ANXA1^−/−^ +HFD.

**Figure 8:**
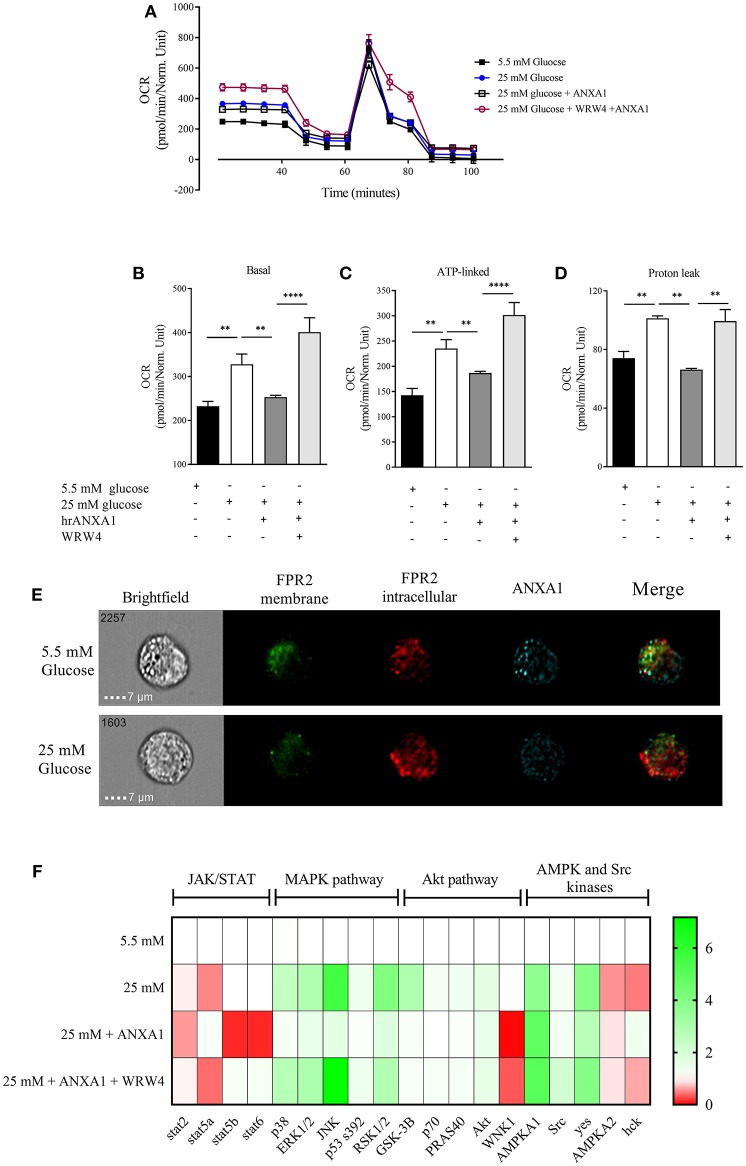
ANXA1 protects human hepatocytes from excess proton leak via FPR2. HepG2 cells were grown for 48 h in 5.5 mM glucose, 25 mM glucose, 25 mM glucose + hrANXA1, or 25 mM glucose + hrANXA1 + WRW4. **(A)** Oxygen consumption rate was assessed using SeaHorse analyzer. **(B)** Basal OCR, and **(C)** ATP-linked OCR, and **(D)** proton leak. **(E)** Expression and localization of FPR2 and ANXA1 assessed by immunofluorescent staining and visualized using image stream. **(F)** 1 × 10^6^ HepG2 cells were grown in 6 well-plates and protein isolated to assess phosphorylation state of 43 proteins using human phospho-proteome profiler. **(B–D)** Data were analyzed by a one-way ANOVA followed by a Bonferroni *post-hoc* test and the mean is expressed mean ± SEM., ^**^p < 0.01, ^****^p < 0.0001 vs. HepG2 + 25 mM glucose. **(F)** Data expressed as fold change to HepG2 + 5.5 mM glucose of pooled samples from 3 independent experiments.

All data in the text and figures are presented as mean ± standard error mean (SEM) of n observations, were n represents the number patients per group (Figures 1,2), animals studied (Figures 3**–7**) or technical replicates (**Figure 8**). All statistical analysis was calculated using GraphPad Prism 7 for Mac (GraphPad Software, San Diego, California, USA). Data without repeated measurements was assessed by a one-way ANOVA followed by Bonferroni correction. Some of the human data were analyzed by Student's *t*-test (data with normal distribution) or Mann Whitney *U*-test (data that were not normally distributed). Correlation studies were analyzed by linear regression using Fishers *F*-test. In all cases a *p* < 0.05 was deemed significant.

## Results

### Patients With Type-2 Diabetes Have Elevated Plasma Levels of ANXA1 Correlated With Increased Dyslipidemia and Fatty Liver Index

To gain a better understanding of the role of ANXA1 in man, we compared the plasma levels of ANXA1 in gender- and age-matched subjects with normoglycemia or T2D (Table 1). Of note, patients with T2D had significantly higher circulating plasma levels of ANXA1 (Figure 1A) when compared with normoglycemic subjects. Moreover, the plasma levels of ANXA1 in patients with T2D correlated positively with fatty liver index, a surrogate marker of hepatic lipid accumulation (Figure 1B), and serum LDL-C and total cholesterol (Figures 1C,D), thus implying a relation between elevated circulating ANXA1 levels and a complex metabolic pattern, including altered glucose control, hepatic steatosis and dyslipidemia. As expected, CRP (a marker of chronic low-grade inflammation) correlated positively with increased BMI in our cohort of patients with T2D (Figure 2A). Interestingly circulating plasma ANXA1 did not correlate with either BMI or CRP (Figures 2B,**C**). Additionally, we observed no correlation of CRP and lipid levels (Figures 2D,E). Additionally, ANXA1 levels were not further elevated in patients who presented with diabetes and CKD (Figure 2F). This result is particularly interesting, as a heightened level of systemic inflammation is classically seen in patients with CKD. Taken collectively, these observations suggest that the observed increase in plasma ANXA1 is not a consequence of increased systemic inflammation, but secondary to the altered metabolic state and aberrant lipid handling.

**Table 1:** Detail of patients included in the study.

	**Normoglycemic**	**T2D**
Number (n)	30	65
Age (years)	71 ± 0.4	69 ± 0.9
Gender (F%)	13 (43)	46 (35)
Fasting glucose (mg/dL)	101.3 ± 1.2	171.7 ± 5.6^*^
Hb1Ac (%)	N/A	7.37 ± 0.1
BMI (kg/m^2^)	26.3 ± 0.6	31.4 ± 0.8^*^
Waist Circumference	91.3 ± 1.5	105.91 ± 1.74^*^
Total cholesterol (mg/dL)	189.5 ± 4.8	187.27 ± 5.48
HDL-C (mg/dL)	59.7 ± 1.7	48.02 ± 1.85^*^
LDL-C (mg/dL, Friedewald)	109.7 ± 4.3	109.61 ± 4.70
Triglycerides (mg/dL)	100.1 ± 5.3	148.26 ± 17.4^*^
C-Reactive Protein (mg/dL)	0.12 ± 0.02	0.58 ± 0.2^*^
Alanine Aminotransfersae (ALT) (U/L)	19.54 ± 0.96	31.89 ± 4.8^*^
Aspartate Aminotransferase (AST) (U/L)	24.08 ± 0.61	26.71 ± 3.1^*^
Gamma Glutamyl Transpeptidase (GGT) (U/L)	28.6 ± 3.15	35.16 ± 2.7^*^
Fatty Liver Index (%)	37.1 ± 5.7	66.14 ± 3.31^*^
Creatinine (mg/dL)	0.9 ± 0.04	1.8 ± 0.1^*^
GFR (mL/min)	71.3 ± 2.34	29.7 ± 1.8^*^
Urinary albumin (mg/g urinary protein)	N/A	202.0 ± 48.1

Number of patients per group, age, gender, fasting glucose, Hb1Ac, BMI, waist circumference serum, cholesterol, HDL, LDL-C, triglyceride, C-reactive protein (CRP), ALT, AST, GGT, FLI, creatinine, GFR, and urinary albumin. Experimental groups: Normoglycemic (n = 30) and patients with type-2 diabetes T2D, (n = 65). Data analyzed by unpaired Student's t-test (normally distributed data) or Mann Whitney U-test (data that are not normally distributed) and expressed as mean ± SEM.

* *p < 0.05; NA, data not available*.

### ANXA1 Attenuates the Development of Obesity and Insulin Resistance in HFD Fed Mice

To gain a better understanding of the role of ANXA1 in the pathophysiology of T2D, we used a murine model of HFD-induced insulin resistance, hepatic steatosis and renal dysfunction (diabetic nephropathy). Consistent with the observation in patients with T2D, mice fed a HFD had elevated levels of circulating ANXA1 (Figure 3A). We also show that the ANXA1 protein levels were dramatically reduced in key target tissues (liver, kidney, and skeletal muscle) of animals fed a HFD (Figure 3B) compared to chow fed mice.

When compared to chow-fed mice, WT-mice fed a HFD gained more weight (Table 2), had elevated levels of serum insulin, higher (non-fasted) blood glucose levels and a significant impairment in tolerance to oral glucose challenge (oral glucose tolerance test, OGTT) (Figures 2C–G). ANXA1^−/−^ fed a HFD gained significantly more weight (Table 2), had higher blood glucose levels (Figure 3D) and an even more severely impaired OGTT (Figures 3F,G) when compared to HFD-fed WT-mice. HFD-fed WT-mice treated with hrANXA1 had normal tissue levels of ANXA1 (Figure 3B), lower serum insulin levels, lower (non-fasted) blood glucose levels (Figures 3C,D) and an improved OGTT (Figures 3E,G) when compared to HFD-fed WT-mice. Administration hrANXA1 to ANXA1^−/−^ mice (rescue experiment) resulted in significantly lower serum insulin levels, blood glucose levels, a reduction in weight gain and an improvement in OGTT; suggesting that endogenous ANXA1 is a key mediator of glucose homeostasis.

**Table 2:** Animals biochemistry.

		**Wild**	**Type**			**ANXA1 -/-**	
	**Chow**	**Chow + hrANXA1**	**HFD+ Vehicle**	**HFD + hrANXA1**	**Chow**	**HFD + Vehicle**	**HFD + hrANXA1**
Body weight (g)	9.790 ± 0.602^*^	9.857 ± 0.674^*^	16.81 ± 0.479	12.87 ± 0.455^*^	4.550 ± 0.276^$^	13.89 ± 0.772	7.00 ± 0.447^$^
Epididymal fat (mg)	1.963 ± 0.163^*^	2.100 ± 0.255^*^	3.570 ± 0.291	2.867 ± 0.253^*^	2.288 ± 0.067^$^	5.609 ± 0.246	4.361 ± 0.007^$^
Inguinal fat (mg)	1.250 ± 0.128^*^	1.275 ± 0.149^*^	2.320 ± 0.243	1.833 ± 0.212^*^	0.714 ± 0.051^$^	1.842 ± 0.096	1.370 ± 0.094^$^
Blood glucose (mmol/L	8.438 ± 0.541^*^	7.682 ± 0.29^*^	13.86 ± 0.6621	9.088 ± 0.442^*^	8.650 ± 0.493^$^	19.56 ± 1.224	8.483 ± 0.541^$^
Serum Insulin (ng/mL)	6.6941.282^*^	7.110 ± 1.061^*^	18.73 ± 2.454	7.860 ± 0.697^*^	5.321 ± 0.238^$^	20.54 ± 1.271	13.47 ± 0.817^$^
OGTT (AUC)	84.42 ± 4.506^*^	84.96 ± 3.659^*^	110.7 ± 3.659	89.42 ± 0.999^*^	87.65 ± 4.716^$^	127.6 ± 3.767	99.93 ± 3.560^$^

Body weight gain from baseline, epididymal fat weight, inguinal fat weight, blood glucose, serum insulin, and OGTT AUC were measured in WT C57BL/6 or ANXA1^−/−^ mice fed a standard diet (chow) or a high-fat diet (HFD) for 10 weeks. HFD-mice were treated with either vehicle or human recombinant (hr) ANXA1 (40 μg/kg, i.p.) five times per weeks between weeks 4 and 10. Data are expressed as mean± SEM of 7–10 mice per group. Data were analyzed by a one-way ANOVA followed by a Bonferroni post-hoc test,

* p < 0.05 vs. WT + HFD or

$ *p < 0.05 vs. ANXA1 + HFD*.

### ANXA1 Improves IRS-1 Signal Transduction in HFD-Induced Insulin Resistance

As endogenous ANXA1 protected against the development experimental diabetes and treatment with hrAXNXA1 improved the diabetic phenotype of HFD-fed mice, we next investigated the potential mechanisms underlying the observed beneficial effects of both hrANXA1 and endogenous ANXA1.

When compared to WT-mice fed a chow diet, WT-mice fed a HFD exhibited an increase in the degree of phosphorylation of insulin substrate receptor-1 (IRS-1) on Ser^307^ in skeletal muscle (Figure 4A) and liver (Figure 4D); as well as a decrease in the phosphorylation of downstream effectors of the insulin signaling pathway, protein kinase B (Akt) on Ser^473^ (Figures 3B,E) and glycogen synthase kinase-3β (GSK-3β) on Ser^9^ in both skeletal muscle and liver (Figures 4C,F). All of the above findings suggest that WT-mice fed a HFD had developed peripheral insulin resistance. ANXA1^−/−^ mice fed a HFD exhibited a further significant increase in the degree of phosphorylation of IRS-1 on Ser^307^ in both skeletal muscle and liver (Figures 4A,D); resulting in a decrease in the phosphorylation of Akt on Ser^473^ (Figures 4B,E) and glycogen synthase kinase-3β (GSK-3β) on Ser^9^ (Figures 4C,F). These data are consistent with the more severe diabetic phenotype observed in ANXA1^−/−^ mice fed a HFD.

Treatment of WT-mice fed a HFD with hrANXA1 attenuated the increase in phosphorylation of IRS-1 on Ser^307^, and the subsequent decrease in phosphorylation of Akt on Ser^473^ and GSK-3β on Ser^9^ in both skeletal muscle (Figures 4A–C) and liver (Figures 4D–F). Additionally, when hrANXA1 was given to ANXA1^−/−^ mice (rescue experiment), all abnormal signaling events were restored to that of WT mice fed a chow diet and highlighting of ANXA1 as a signaling molecule in the IRS-1 signal transduction pathway.

### ANXA1 Attenuates Dyslipidemia, Steatosis, Liver Injury, and Renal Dysfunction in HFD Fed Mice

In addition to developing insulin resistance, WT-mice fed a HFD had increased levels of serum triglycerides, cholesterol, a 10-fold increase in liver triglyceride levels (Figures 5A–C) associated with an increase in Oil Red-O staining in the liver (Figure 5G), suggesting the development of dyslipidemia and liver steatosis. Lipid deposition and steatosis in the liver is associated with liver injury; WT-mice fed a HFD had elevated serum ALT levels (Figure 5D) compared to WT mice fed a chow diet. ANXA1^−/−^ mice fed a HFD had even significantly higher serum triglyceride levels, further increased Oil Red-O staining in the liver and even higher ALT levels when compared to WT mice fed a HFD (Figures 5A–D,G). In contrast, WT-mice fed a HFD and treated with hrANXA1 had significantly lower serum cholesterol and liver triglyceride levels, less Oil Red-O staining in the liver and significantly lower ALT levels (Figures 5A–D,G) compared to HFD-fed WT-mice. Treatment of ANXA1^−/−^ mice with hrANXA1 (rescue experiment) resulted in a reduction in liver triglyceride levels, serum ALT, and Oil Red-O accumulation in the liver compared to ANXA1^−/−^ mice fed a HFD (Figures 5A–D,G). Taken together, these findings suggest that ANXA1 protects against the development of dyslipidemia and liver steatosis/injury.

T2D ultimately drives the development of microvascular complications including diabetic nephropathy. WT-mice fed a HFD exhibited an elevated albumin-to-creatinine ratio (ACR) and decreased creatinine clearance (Figures 5E,F) compared to chow-fed WT-mice, suggesting the development of proteinuria (a marker of diabetic nephropathy) and renal dysfunction. The development of proteinuria was associated with significant morphological changes. WT-mice fed a HFD have more vacuolar degeneration at the level of the S1-S2 segment of the proximal convoluted tubules, while other tubular structures of the nephron remained histologically preserved (Figure 5H). There was also a marked loss of brush boarders in the S1-S2 segment of the proximal convoluted tubules (yellow arrows). All histological markers of tubular degeneration were more marked in the kidneys from ANXA1^−/−^ mice fed on a HFD (Figure 4H). Which correlated with ANXA1^−/−^ mice fed a HFD having further elevation in ACR (Figure 5E), suggesting the development of more severe proteinuria. In contrast, administration of hrANXA1 to HFD-fed WT-mice attenuated the rise in ACR and the decrease creatinine clearance (Figures 5E,F) and preserved the kidney from signs of tubular degeneration (Figure 5H).

### ANXA1 Regulates eNOS and RhoA Activation in a Model of HFD-Induced Insulin Resistance

One of the key drivers of proteinuria and, indeed, renal dysfunction is renal hypertension, which is, in part driven by the development of endothelial dysfunction. WT-mice fed a HFD had a significant reduction in the phosphorylation of eNOS on Ser^1177^ in the kidney (Figure 6) compared to chow-fed WT animals; while hrANXA1 treatment significantly prevented the reduction in phosphorylation of eNOS on Ser^1177^ (Figure 6). Moreover, ANXA1^−/−^ mice fed a HFD also demonstrated significantly reduced phosphorylation of eNOS on Ser^1177^. Interestingly, ANXA1^−/−^ mice fed with HFD and treated with hrANXA1 (rescue experiment) had no changes in the phosphorylation of eNOS on Ser^1177^ (Figure 6), indicating that hrANXA1 prevents the decline in eNOS phosphorylation seen in ANXA1^−/−^ mice fed with HFD.

Having shown that endogenous ANXA1 limits, and that administration of hrANXA1 attenuates the development of peripheral insulin resistance as well as liver injury and kidney dysfunction caused by a HFD, we further explored the potential mechanism(s) underlying the observed beneficial effects of ANXA1. Activation of RhoA plays a key role in the development of both peripheral insulin resistance and in the development of microvascular complications of diabetes (9). We have previously demonstrated that ANXA1 interacts with RhoA (7). Here we demonstrate that the tissue levels of ANXA1 are decreased in mice fed a HFD (Figure 2B). This resulted in reduced phosphorylation of RhoA on Ser^188^ (increased GTPase activity) and in an increase in the phosphorylation of downstream effector MYPT1 on Thr^853^ in the kidney (Figures 7A,D), liver (Figures 6B,E), and skeletal muscle (Figures 7C,F), when compared to chow-fed WT-mice.

Interestingly, ANXA1^−/−^ mice also have a reduced phosphorylation of RhoA on Ser^188^ (Figures 7A–C), and an increased phosphorylation of MYPT1 on Thr^853^ (Figures 7D–F) (regardless of dietary manipulation) in kidney and skeletal muscle, suggesting that ANXA1^−/−^ mice have constitutively activated RhoA-GTPase. Treatment of ANXA1^−/−^ mice fed a HFD with hrANXA1 (rescue experiment) resulted in an increase in the degree of phosphorylation of RhoA on Ser^188^ (Figures 7A–C) and attenuation of the degree of phosphorylation on MYTP1 on Thr^853^ (Figures 7D–F), in liver, kidney, and skeletal muscle; demonstrating conclusively that ANXA1 regulates RhoA activity.

From a therapeutic point of view, treatment of WT HFD-fed mice with hrANXA1, also restored the tissue levels of ANXA1 in liver, kidney, and skeletal muscle (Figure 2B). This prevented the reduction of the phosphorylation of RhoA on Ser^188^ (Figures 7A–C), and the associated increase in phosphorylation of MYPT1 on Thr^853^ (Figures 7D–F) observed in HFD-fed WT-mice. These findings support the view that ANXA1 regulates the activity of RhoA in experimental diabetes, which, in turn, limits the development of peripheral insulin resistance and protects the kidney and liver from functional decline.

### ANXA1 Protects Against Excessive Proton Leak via FPR2 in Human Hepatocytes

One of the major sites of both lipid and glucose handling is the liver. Therefore, we wanted to investigate whether ANXA1 is involved in the regulation of energy homeostasis in human hepatocytes. Therefore, in order to evaluate whether hepatic mitochondrial respiration was altered under high glucose conditions, oxygen consumption rate (OCR) was measured (Figure 8A). HEPG2 cells grown in high glucose medium had higher basal OCR (Figure 8B), increased ATP-linked OCR (Figure 8C) leading to excessive proton leak (Figure 8D), which can lead to the formation of reactive oxygen species and cellular damage. When HEPG2 cells were incubated in high glucose (25 mM) medium in the presence of hrANXA1, hrANXA1 attenuated the increases in basal OCR (Figure 8B), ATP-linked OCR (Figure 8C), and proton leakage (Figure 7D). We hypothesized that the observed beneficial effects of treatment with hrANXA1 are mediated though the FPR2 receptor. Interestingly, expression levels of FPR2 was not altered by exposure of HEPG2 cells to high glucose (25 mM) medium, whereas ANXA1 levels were decreased (Figure 8E). Using a specific FPR2 antagonist, we were able to block the effects of hrANXA1 on basal OCR (Figure 8B), ATP-linked OCR (Figure 8C), and proton leak (Figure 8D) suggesting the beneficial effects of hrANXA1 in reducing excessive proton leak are, indeed, FPR2 mediated.

### Effects of hrANXA1 on MAPK and Akt Pathway Are FPR2 Mediated in Human Hepatocytes

To get a better understanding of the signaling pathways modulated by hrANXA1 in HepG2 cells during exposure to high glucose, the phosphorylation states of 43 potentially relevant pathways were measured using a human phospho-kinase proteome profiler array (Figure 8F and Figure S1). Our results demonstrate that HepG2 cells grown in high glucose medium (25 mm) had an increase in phosphorylation of key members of the MAPK pathway (p38, JNK, ERk1/2, and RSK1/2) and in the Akt pathway (Akt, GSK-3B, p70, and PRAS40), while key members of the pro-inflammatory JAK/STAT pathway exhibited decreased inhibitory phosphorylation (Stat2 and Stat5a). When HepG2 cells were grown in high glucose medium and treated with hrANXA1, the observed increases seen in the phosphorylation of the MAPK (p38, JNK, ERk1/2, and RSK1/2) and the Akt pathway (GSK-3B, p70, and PRAS40) were attenuated. Additionally, AMPKa2 phosphorylation was further increased in the presence of hrANXA1. Pre-incubation with an FPR2 antagonist abolished all of the above effects of treatment with hrANXA1, strongly suggested that ANXA1 is signaling through FRP2 (Figure 8F).

## Discussion

The key findings of our study are that patients with T2D have elevated plasma ANXA1 levels, which correlate positively with fatty liver index and serum lipid levels, but not with markers of systemic inflammation, suggesting that the ANXA1 levels in patients with T2D are not regulated by systemic inflammation, but are a consequence of aberrant lipid handling. To elucidate the role of ANXA1 in the pathophysiology of obesity/metabolic syndrome, we used a model of HFD-induced insulin resistance in WT and ANXA1^−/−^ mice. Consistent with our patient data, mice fed a HFD showed elevated serum ANXA1 levels, while their tissue levels were reduced (see below). ANXA1^−/−^ mice fed a HFD developed a more severe diabetic phenotype (compared to WT mice) as indicated by higher fasting glucose levels, increased impairment in the oral glucose tolerance test and increased impairment of insulin signaling (IRS-1, Akt, and GSK-3β). Additionally, treatment of WT-mice fed a HFD with hrANXA1 reduced the diabetic phenotype, restored IRS-1 and Akt (liver and skeletal muscle) and eNOS (kidney) activity. ANXA1^−/−^ mice fed on a HFD also developed severe dyslipidemia, hepatosteatosis (lipid accumulation in the liver) and renal dysfunction (proteinuria), while the therapeutic administration of hrANXA1 attenuated both hepatosteatosis and the renal dysfunction/proteinuria caused by HFD. HFD-fed mice had lower intracellular levels of ANXA1 in the kidney, liver and skeletal muscle resulting in the activation of small GTPase RhoA and of the downstream effector MYTP1: This key finding was confirmed in ANXA1^−/−^ mice, which had constitutively activative RhoA. Treatment with hrANXA1 restored intracellular ANXA1 levels, reduced RhoA activation and restored IRS-1 signaling. Thus, we demonstrate here for the first time a mechanistic link between ANXA1 levels, RhoA activity and IRS-1 signaling. Treatment of human hepatocytes with hrANXA1 also reduced the mitochondrial proton leak in an FPR2-dependent manner. All of the above findings support the conclusions that endogenous ANXA1 prevents the development of insulin-resistance and associated microvascular complications, while pharmacologically administered hrANXA1 attenuates the development of metabolic and secondary microvascular complications in experimental T2D.

What, then, is the mechanism(s) by which ANXA1 attenuates insulin resistance in experimental T2D and protects against the development of secondary complications? The observed impairment in glucose tolerance seen in mice fed on a HFD is, in part, mediated by alterations in insulin signaling in liver and skeletal muscle. The IRS-1/Akt/GSK-3β signaling cascade is a key regulator of glucose transportation, glycogen synthesis, and energy metabolism (glycolysis) (17). Peripheral insulin resistance is attributed to an increase in the phosphorylation of Ser^307^ on IRS-1; this uncouples IRS-1 from the insulin receptor blunting the ability of insulin to signal through its receptor, which, in turn, reduces GLUT4 translocation to the cell surface to facilitate glucose uptake in peripheral organs, leading to hyperglycemia (18). Mice fed a HFD showed increased phosphorylation of Ser^307^ on IRS-1 (in the liver/skeletal muscle), which was further augmented in ANXA1^−/−^ mice. In contrast, treatment with hrANXA1 attenuates this increase in the phosphorylation of Ser^307^ on IRS-1 resulting restoration/maintenance of normal glucose levels in the blood (19).

Many of the beneficial effects of anti-diabetic drugs are mediated through inhibition of the GTPase RhoA. Metformin reduces RhoA activity and activates AMPK (20, 21), while Fasudil, a selective RhoA inhibitor used to treat diabetic nephropathy, improves insulin signaling and nephropathy by correcting glucose and lipid homeostasis in obese Zucker rats (22–24). Statins, which are best known for their lipid-lowering effects, also ameliorate the progression of diabetic nephropathy, and both effects have been attributed to inhibition of RhoA/ROCK (25–29). Interestingly, RhoA activation is inhibited by ANXA1 (7), suggesting an original mechanistic link between ANXA1 levels, RhoA activation and the development of insulin resistance. We demonstrate that mice fed a HFD have an increase in the circulating levels of ANXA1, but this was associated with reduced expression in skeletal muscle, liver, kidney, which (in turn) resulted in activation of RhoA. However, we do not know whether the circulating ANXA1 measured in animals and man is inactive or whether the subsequent treatment restores the loss of the biological activity; though we need to take in consideration the limited comparability of the mouse model vs. the human condition. Moreover, we also demonstrate that ANXA1^−/−^ mice have constitutively activated RhoA activity suggesting that ANXA1 is a regulator of RhoA activity. Thus, we propose that endogenous ANXA1 is a regulator of RhoA/ROCK activity under physiological conditions. Indeed, treatment of HFD-fed mice with hrANXA1 restored intracellular ANXA1 levels/signaling (Figure 2B), modulated RhoA phosphorylation and re-stabilized the insulin receptor (resulting in reduced phosphorylation of IRS-1) allowing restoration of insulin signaling and glucose homeostasis. Cristante et al. (7) have previously demonstrated in the brain microvascular endothelium that ANXA1 and RhoA co-precipitate together suggesting a direct molecular interaction. Here we demonstrate for first time a mechanistic link between ANXA1, RhoA, and IRS-1 signaling *in vivo*.

Impairments in insulin sensitivity are associated with excessive lipid deposition (18). WT mice fed on a HFD developed dyslipidemia, had excessive fat accumulation in their peripheral fat beds (Table 1) and in the liver causing hepatic steatosis and liver injury (elevated ALT levels), all of which were more severe in ANXA1^−/−^ mice. In contrast, mice fed a HFD and treated with hrANXA1 show reduced adiposity, and a reduction in lipid and triglyceride accumulation in the liver. Collectively, our findings suggest that ANXA1 reduces the development of hepatic steatosis and the associated liver injury by inhibition of RhoA activity and restoration of IRS-1 signaling. Indeed, our human data shed further light on the role of ANXA1 in lipid homeostasis, as ANXA1 plasma levels correlate positively with increased lipids in both the liver and the circulation, but do not correlate with systemic inflammation, suggesting a new biological function of ANXA1 beyond those of as an anti-inflammatory mediator.

We also report here that mice fed a HFD have reduced phosphorylation (Ser^473^) and, hence, reduced activation of Akt. Activated Akt regulates inflammatory and pro-survival responses (30). We also show in human hepatocytes that treatment with ANXA1 re-activated Akt in an FPR2-dependent manner. One consequence of inhibition of Akt is that organs are less resistant to stressor stimuli including hyperglycemia and/or hyperlipidemia and subsequently develop organ injury (15). In contrast, restoration of the degree of activation of the Akt pathway reduces organ injury in many conditions associated with inflammation including sepsis-induced organ dysfunction (31, 32), hemorrhagic shock-induced organ dysfunction (33, 34), and diabetes (6, 18). The N-terminal peptide of ANXA1 (Ac2-16) also reduces tissue injury by activating Akt in experimental models of cardiac/renal reperfusion injury (35, 36) and non-alcoholic steatohepatitis (37). Activation of Akt also results in inhibition GSK-3β (Ser^9^ phosphorylation) resulting in activation of glycogen synthase, which converts glucose to glycogen for storage in the liver. Here we demonstrate that mice fed on a HFD have decreased activation of GSK-3β, which could result in a reduction in glycogen synthesis and increased blood glucose levels. In contrast, mice treated with hrANXA1 have restored phosphorylation of GSK-3β; allowing for the enzymatic conversion of glucose to glycogen.

We demonstrate using extracellular flux assay that hyperglycemia alter mitochondrial function resulting in the production of excessive protons, that can lead to the production of excessive ROS, which primarily damage the mitochondria. Here we shown that hrANXA1 protects the mitochondria from the detrimental effects of hyperglycemia in an FPR2-dependent manner. Consistent with previous *in vivo* data (6) we show that the treatment of human hepatocytes grown in under high glucose with hrANXA1attenuates the activation of MAPK and restores Akt and GSK-3β activity. Incubation of HEPG2 cells with normal (5.5 mM) results in co-localization of ANXA1 and FPR2 on the plasma membrane. In contrast, treatment of these cells with high glucose (25 mM) results in a clear separation of the localization of ANXA1 and FPR2 (Figure 8). Such data clearly suggests that high glucose conditions effects ANXA1 distribution and its inability to work in an autocrine way (38).

Insulin resistance results in the development of microvascular complications including diabetic nephropathy. ANXA1^−/−^ mice fed a HFD have more severe renal dysfunction (proteinuria and a reduction in GFR) when compared to WT mice fed on a HFD diet. Patients with T2D display endothelial dysfunction, which is associated with an increased cardiovascular risk (39). Several lines of evidence suggest that decreased eNOS phosphorylation is a molecular mechanism linking aberrant metabolism and vascular dysfunction: (i) eNOS phosphorylation is diminished in diabetes, hypercholesterolemia, and atherosclerosis (40–42), (ii) anti-diabetic drugs including statins and PPAR agonists increase eNOS phosphorylation (37), and (iii) signaling molecules including insulin, IGF-1, and leptin increase eNOS phosphorylation (39). Here we demonstrate that mice fed on a HFD have decreased eNOS phosphorylation (in the kidney), which was restored by hrANXA1 treatment. The present study shows that mice fed a HFD have decreased Akt and eNOS activity, while both of these signaling events are attenuated by treatment with hrANXA1. Indeed, inhibition of RhoA/ROCK by statins or other selective inhibitors leads to the up regulation and activation of eNOS (43), potentially through restoration of Akt signaling (39). We also demonstrate that inhibition of RhoA attenuates the activity of MYTP1. MYPT1 is a key regulator of vascular tone within vascular smooth muscle. Activated (phosphorylated) MYPT1 actively phosphorylates myosin delaying its ability to relax, therefore, inducing prolonged contraction (44, 45) suggesting there could be a link between ANXA1 and the development of hypertension (however this warrants further investigation).

We report here for the first time that plasma levels of AXNA1 are also elevated in patients with T2D, thus supporting the hypothesis that increased circulating ANXA1 levels are indicative of an immune-metabolic state. Obesity is one of the strongest etiological predictors for developing T2D and of adverse cardiovascular outcomes. Here we clearly demonstrate a positive correlation between increased hepatic steatosis (fatty liver index), waist circumference (Table 1) and elevated plasma ANXA1 levels (Figure 1A); suggesting that high ANXA1 levels may also be a biomarker of the development of fatty liver disease (although this hypothesis warrants further investigation). Dyslipidemia is a key predictive factor for both the development of T2D and adverse cardiovascular events. Here we show for the first time a strong positive correlation between elevated total cholesterol and LDL-C and elevated plasma ANXA1 levels. Critically, ANXA1 levels were not correlated with CRP and marker of chronic inflammation. This is consistent with our previous finding showing that ANXA1 levels did not correlate with CRP in patients with type-1 diabetes. We hypothesize that ANXA1 is released from cellular stores into the circulation in response to an aberrant physiological conditions (6) where it is known to signal in both an autocrine and paracrine manner through Formyl-Peptide receptors to elicit both anti-inflammatory and tissue protective effects (5). Two independent lines of evidence support this (1) ANXA1 levels are elevated in both patients and mice with T2D and (2) tissue levels of ANXA1 are decreased in HFD-fed mice (Figure 1) suggesting it has been released from intracellular stores.

## Conclusions

In conclusion, we report here for the first time that mice fed a HFD develop insulin resistance, while endogenous ANXA1 dampens the development of both the diabetic phenotype and the associated hepatic steatosis and nephropathy (proteinuria). Most notably, treatment with hrANXA1 re-establishes normal insulin signaling and attenuates the development of hepatosteatosis and diabetic nephropathy. ANXA1 regulates and inhibits RhoA activity *in vivo*. In murine diabetic tissues, expression of ANXA1 is decreased allowing for the activation of RhoA, while treatment with hrANXA1 inhibits RhoA activity, decreases insulin resistance, and restores Akt and eNOS signaling. Finally, we demonstrate that patients with T2D have elevated plasma levels of ANXA1. Thus, we propose that elevated levels of ANXA1 may represent a novel biomarker for the development of hepatosteatosis and that hrANXA1 or its peptide mimetics may be useful in the treatment of T2D and/or its complications.

## Author Contributions

GP, MC, AB, GN, CT, and ES drafted the manuscript and provided important intellectual content. RAL performed Seahorse and image stream analysis. FC, DC, AC, RM, MA, and JC acquired data on signaling. MB and MS acquired Elisa data. LG, ALC, and CR provided human sample collection and clinical data set and discussion. CR expressed and purified the human recombinant ANXA1, MY contributed to the discussion. GP, CT, and ES are the guarantors of this work. All authors made substantial contributions to conception and design, acquisition of data and interpretation of data, reviewed, and approved the manuscript.

### Conflict of Interest Statement

The authors declare that the research was conducted in the absence of any commercial or financial relationships that could be construed as a potential conflict of interest.

## Supplementary Material

Supplementary Figure 1 1 × 10^6^ HepG2 cells were grown for 48 h in 5.5 mM glucose, 25 mM glucose, 25 mM glucose + hrANXA1, or 25 mM glucose + hrANXA1 + WRW4. Protein was isolated to assess phosphorylation state of 43 proteins using human phospho-proteome profiler, 23 are reported in the figure. Data expressed as fold change to HepG2 + 5.5 mM glucose of pooled samples from 3 independent experiments.
