# Supplementary figures and images for: Identification of AnnexinA1 as an Endogenous Regulator of RhoA, and Its Role in the Pathophysiology and Experimental Therapy of Type-2 Diabetes
Source: Front Immunol. 2019 Mar 27;10:571. doi: 10.3389/fimmu.2019.00571 (PMC6446914; doi:10.3389/fimmu.2019.00571)

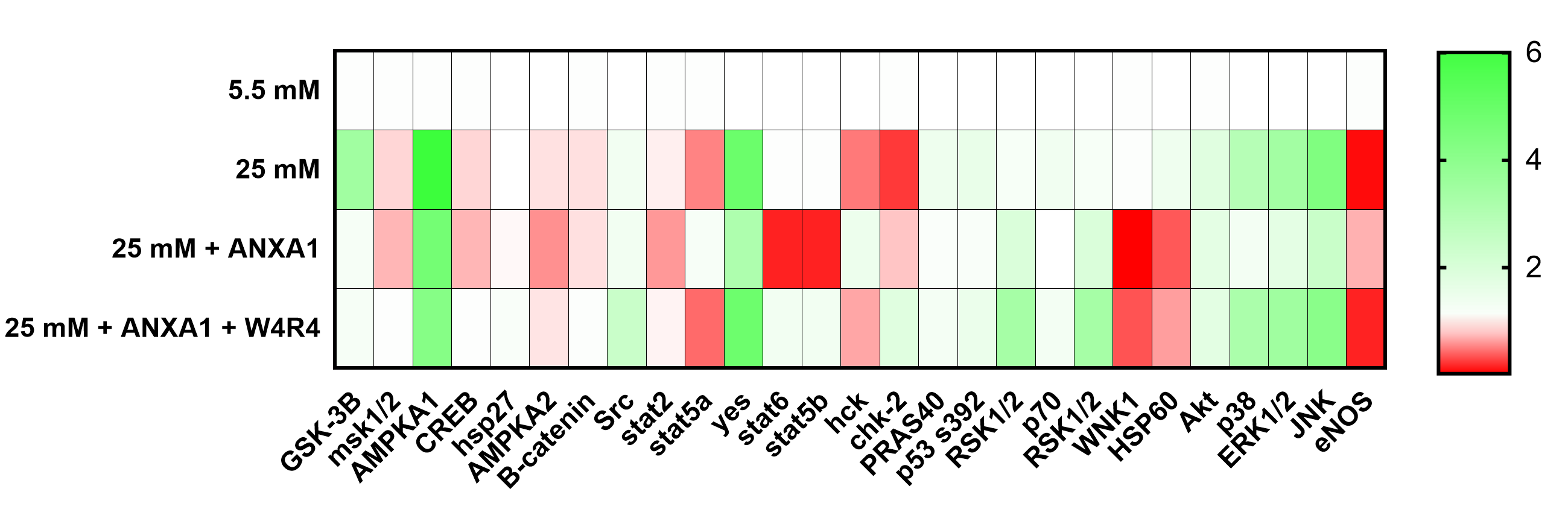

Supplement: Supplementary Figure 1 — 1 × 10^6^ HepG2 cells were grown for 48 h in 5.5 mM glucose, 25 mM glucose, 25 mM glucose + hrANXA1, or 25 mM glucose + hrANXA1 + WRW4. Protein was isolated to assess phosphorylation state of 43 proteins using human phospho-proteome profiler, 23 are reported in the figure. Data expressed as fold change to HepG2 + 5.5 mM glucose of pooled samples from 3 independent experiments. [file Image_1.TIF]
